# Advances in Quantitative Mass Spectrometry

**DOI:** 10.1155/2013/621029

**Published:** 2013-04-29

**Authors:** Mu Wang, Bomie Han, Valerie Wasinger

**Affiliations:** ^1^Department of Biochemistry and Molecular Biology, Indiana University School of Medicine, Indianapolis, IN 46202, USA; ^2^Tailored Therapeutics-Mass Spectrometry, Lilly Research Laboratories, Eli Lilly & Company, Indianapolis, IN 46285, USA; ^3^Bioanalytical Mass Spectrometry Facility, The University of New South Wales, Sidney, NSW 2052, Australia

Mass spectrometry (MS) has played an ever-increasing role in the basic sciences since the invention of ESI, trap, and MALDI techniques. MS has been applied extensively in genomics, proteomics, transcriptomics, and metabolomics for biomolecular identification, quantification, and characterization, making it a powerful tool for the analysis of biological processes and biomarker development. In particular, the application of quantitative mass spectrometry offers new opportunity and great potential to develop innovative diagnostic and prognostic tests, to identify novel therapeutic targets, to allow the design of individualized patient treatment, and eventually to extend healthy life and reduce the burdens of illness and disability. 

In recent years, our capabilities in the “omics” field have been profoundly enhanced due to the advances in both hardware and software. The performance capabilities, easy operation, and robustness of MS over other techniques have made it an ideal platform for quantitative proteomics. This platform offers an analytical tool for faster, higher throughput, and wider dynamic range protein analysis and can be applied in both stable-isotope labeled or label-free manner for relative and absolute quantitation of proteins and peptides in vastly complex samples. 

The advancement in quantitative mass spectrometry is reflected by the wide range of topics covered in this special issue. 

H. V. Trinh et al. compare quantitative results using labeled (iTRAQ) and label-free (ion count) approaches to study the proteomes of noninfected and human adenovirus infected cells. In this study, multiple informatics quantitation tools were evaluated.

V. Wasinger et al. provide comprehensive and critical overview of quantitative techniques used in biomarker research from the low-cost discovery-driven to hypothesis-driven quantitation using synthetic standards with complimentary analysis of trends by alternative techniques. A comparative overview with associated informatics links provides an excellent snapshot of proteomic quantitative techniques. 

Z. Liao et al. present novel use of SILAC method to generate internal standard peptides for the kinetic study of protein synthesis and degradation to reveal potential linkage between vacuolar protein sorting 4B (VPS4B) and fatty acid *β*-oxidation pathway in breast cancer cells.

O. Anania et al. illustrate a novel peptide-based SILAC method that, in combination with quantitative mass spectrometry, can be utilized to characterize and quantify protein posttranslational modifications, even those with novel modifications that may not be detected using conventional proteomic workflows or informatics algorithms. 

X. Lai et al. present common issues associated with the label-free protein quantification approach including qualification of the peptides, chromatographic peak alignment, peak normalization, and an overview of the method and examples of their use to overcome some of the technical challenges. 

J. E. Hale presents some case-specific examples that compare the utility of MS-based protein quantification technique with immunobased techniques to identify and quantify protein isoforms and PTMs.

R. E. Higgs et al. investigate the use of MS1 quantification from multiple peptides for inference at the protein level while investigating the effect of ion interference and PTMs. By using protein standards in background matrices, methods for combining peptide level quantification for a protein were evaluated.

J. M. Held et al. develop a new approach, MS1 filtering and independent data acquisition, for targeted proteomic analysis. Combining high-resolution proteomics with multiprotease digestion enables quantitative assessment of ErbB2 with excellent reproducibility, improves amino acid sequence and PTM coverage, and decreases assay development time compared to conventional SRM/MRM assays. 

These papers represent an exciting and insightful snapshot of current state of quantitative mass spectrometry. Cutting edge methodologies and their applications, new analytical tools, and future directions of the field are highlighted in this special issue to inspire researchers alike and help advance proteomic research. 

